# Novel concept of routine total arterial coronary bypass grafting through a left anterior approach avoiding sternotomy

**DOI:** 10.1007/s00380-022-02034-x

**Published:** 2022-02-05

**Authors:** Hilmar Dörge, Christian Sellin, Ahmed Belmenai, Silke Asch, Holger Eggebrecht, Volker Schächinger

**Affiliations:** 1 Klinik für Herz- und Thoraxchirurgie, Herz-Thorax-Zentrum, Klinikum Fulda gAG, Pacelliallee 4, 36043 Fulda, Germany; 2 Medizinische Klinik I–Kardiologie, Herz-Thorax-Zentrum, Fulda, Germany; 3 Cardioangiological Center Bethanien (CCB), Frankfurt, Germany

**Keywords:** TCRAT, CABG, Coronary bypass surgery, Coronary artery bypass graft, Minimally invasive surgery

## Abstract

Coronary artery bypass grafting (CABG) via full sternotomy remains a very invasive procedure, often requiring prolonged recovery of the patient. We describe a novel, less invasive approach for totally arterial CABG via a small left anterior thoracotomy in a pilot series of 20 unselected patients. From January to March 2020, 20 consecutive patients (mean age 65.9 ± 9.2 years, 100% male, STS-score: 1.6 ± 2) underwent CABG using only arterial conduits via a small left anterior thoracotomy. Patients were operated on cardiopulmonary bypass with peripheral cannulation and transthoracic aortic cross-clamping. Pulling tapes encircling the great vessels, the arrested empty heart was rotated and moved within the pericardium to enable conventional anastomotic techniques especially on lateral and inferior wall coronary targets. In all patients, left internal mammary artery and radial artery were utilized for bypass with 3.3 ± 0.7 distal coronary anastomoses per patient. Anterior, lateral, and inferior wall territories were revascularized in 100%, 85%, and 70% of patients, respectively. Complete anatomical revascularization was achieved in 95% of patients. ICU stay was 1 day in 17 patients, and 14 of patients left the hospital within 8 days. There was no hospital death, no stroke, no myocardial infarction, and no repeat revascularization. In this pilot series of 20 patients, minimally invasive, totally arterial CABG with avoidance of sternotomy was technically feasible with favorable patient outcomes.

## Introduction

Coronary artery bypass grafting (CABG) remains a very invasive treatment with full sternotomy in the vast majority of patients [1]. Although minimally invasive (MICS) and robotic CABG using lateral approaches significantly reduce invasiveness of the operation, these technique have been performed only in a very few specialized centers in a small number of very selected patients [2–4], probably due to technical complexity and enormous infrastructural prerequisites.

Recently, Babliak et al. [5] proposed a new operative approach for revascularization in multivessel coronary disease via a small left anterior thoracotomy (TCRAT—Total Coronary Revascularization via left Anterior Thoracotomy). We adopted this minimally invasive technique as a routine procedure in our CABG program, whenever feasible. In refinement of the originally proposed technique, we exclusively used arterial grafts. This report summarizes our initial experience with this novel approach in 20 unselected patients, detailing the surgical key steps as well as in-hospital patient outcomes.

## Methods

### Patients

Between January and March 2020, 20 non-selected consecutive patients with multi-vessel coronary disease underwent minimally invasive totally arterial CABG using cardiopulmonary bypass (CPB) and cardioplegic cardiac arrest.

All patients were scheduled after heart team discussion including a recommendation which coronary arteries should be grafted [6] according to guideline indications for elective or urgent isolated surgical revascularization [7]. Emergency patients (meaning same day catheterization and operation) were not offered this approach, otherwise only standard institutional contraindications for conventional CABG were applied. The present report constitutes our initial series of consecutive patients from January to March 2020. Data are part of our internal quality assurance documentation and were retrospectively extracted from patient records and presented as mean (± standard deviation (SD)) or number (percentage), unless otherwise indicated.

### Ethical standards

The study has been approved by the local ethics committee and has therefore been performed in accordance with the ethical standards laid down in the 1964 Declaration of Helsinki and its later amendments. All patients gave their informed consent prior to their inclusion in the study.

### Preoperative evaluation

In addition to preoperative institutional standard examinations, the aorta and major arterial branches were screened with CT scan in all patients for atherosclerotic disease and anatomical abnormalities to plan CPB strategy with peripheral arterial cannulation site either via femoral or axillary vessels, and to ensure safe aortic cross-clamping. Furthermore, special attention was given to the detection of possible subclavian artery stenosis.

Quality and usability of the radial artery (RA) in terms of size, atherosclerotic disease, and anatomical circulatory abnormalities were evaluated with ultrasound, Doppler ultrasound, and with the Allen’s test.

During the study period, two patients were identified preoperatively with contraindication to radial artery harvesting (pathological Allen’s test) and inadequate radial artery size (diameter < 2 mm) or quality (atherosclerotic disease), respectively. They received venous grafts instead of radial artery grafts in addition to LIMA, and they were not included in the study.

### Anesthesia

Standard cardiac anesthesia techniques (i.v. sufentanil 0.5 μg/kg/h, etomidate 0.25 mg/kg, pancuronium 0.1 mg/kg, sevoflurane, propofol 3 mg/kg/h) were used for induction and maintenance of anesthesia, and invasive monitoring was performed with standard arterial and venous lines. A single lumen tube with endobronchial blocker positioned in the left main bronchus was used to initiate single lung ventilation during LIMA harvesting. If single lung ventilation was not well tolerated, LIMA was harvested after CPB has been initiated (see below). All patients had intraoperative transesophageal echocardiography to help position guidewires for femoral arterial and venous cannulation as well as to monitor cardiac function.

### Surgical technique

Patients were operated in a supine position with an inflatable pad under the left chest to facilitate LIMA harvest and heart exposure as necessary. Radial artery harvest was performed minimally invasive using an endoscopic reusable retractor (Bisleri Model, Karl Storz, Tuttlingen, Germany) and a bipolar radiofrequency vessel sealing system (LigaSure, Medtronic, Minneapolis, MN, USA) via a 2.5 cm distal incision at the volar surface of the forearm.

The chest was entered through an anterior muscle-sparing thoracotomy of about 8 cm along the fourth intercostal space. A retractor (Small Thoracotomy Retractor, Delacroix-Chevalier, Paris, France) with 5 cm blades was inserted. Division of intercostal muscles was continued until LIMA was identified and divided. LIMA was harvested under direct surgical vision in a semi-skeletonized technique beyond the origin of left mammary vein using long conventional surgical instruments (35 cm DeBakey forceps and 15 cm electrocautery blade) and a special retractor (MIDAccess IMA Retractor, Delacroix-Chevalier, Paris, France).

Peripheral arterial CPB cannulation was performed according to findings of preoperative CT scan either via right femoral artery (17/19/21 Fr Bio-Medicus, Medtronic, Minneapolis, USA) in 10 patients (50%) or via right axillary artery (16/18 Fr OptiSite Arterial Perfusion Cannula, Edwards Lifesciences, Irvine, USA) in 10 patients (50%), respectively. A small surgical cut-down to expose the anterior surface of the respective artery was performed, and 400 U/kg heparin was administered intravenously prior to insertion of cannulas. A venous cannula (21 Fr Bio-Medicus, Medtronic, Minneapolis, USA) was inserted percutaneously via the femoral vein. An additional venous cannula (15/17 Fr Bio-Medicus, Medtronic, Minneapolis, USA) was inserted into the jugular vein percutaneously if body surface area was greater than 2.0 m^2^ to enhance venous return. Vacuum-assisted venous return was routinely used during CPB to improve heart decompression further. Patients were kept normothermic during CPB.

The pericardium was opened longitudinally from the apex to the ascending aorta and suspended using several stay sutures. The ascending aorta was dissected from the pulmonary trunc and encircled with a tape. Pulling the tape, the ascending aorta was moved toward the thoracic incision into reach of the fingertip to facilitate insertion of a small cannula (7Fr MTAR, Medtronic, Minneapolis, USA). This line was used for infusion of blood cardioplegia and, during cardiac arrest, for venting the left ventricle to decompress the arrested heart further.

A transthoracic aortic clamp (ValveGate DeBakey, Geister, Plymouth, USA) was introduced through the second intercostal space in the anterior axillary line from the left side. Pulling the aortic tape, the aorta was moved toward the thoracic incision and cross clamped under direct vision. Cardiac arrest was induced with infusion of antegrade cold blood cardioplegia (Dr. Franz Köhler Chemie GmbH, Bensheim, Germany) and maintained with intermittent cold re-infusion every 15 min.

**Fig. 1:**
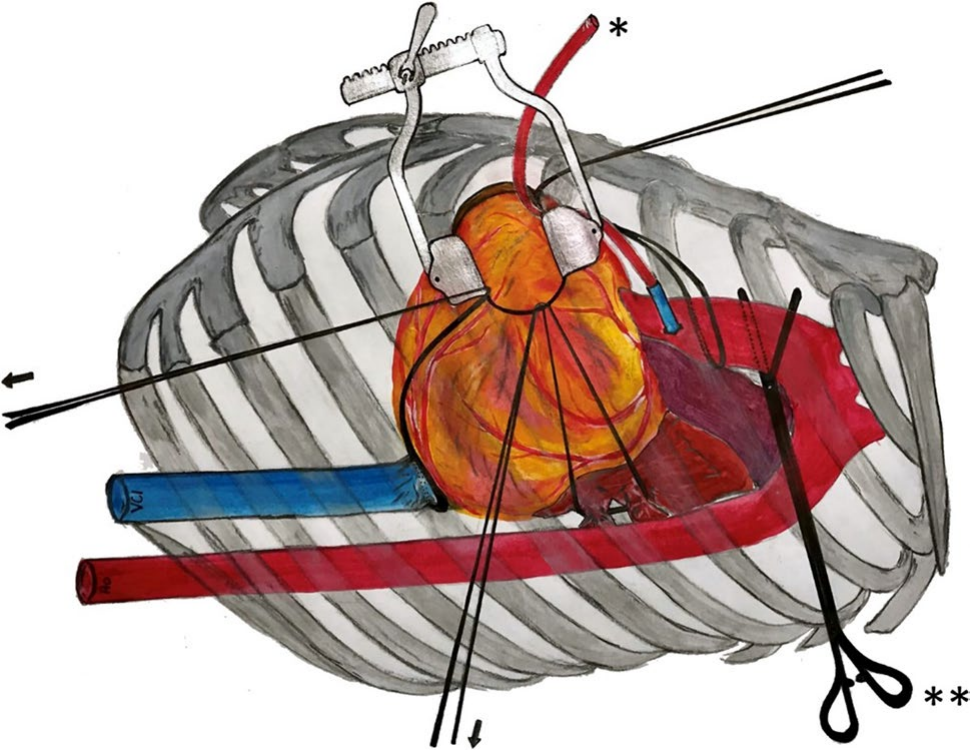
After induction of cardioplegic through cardioplegia line (*), cardiac arrest with transthoracic aortic cross-clamping in the third intercostal space in the left anterior axillary line (**) and peripheral CPB cannulation, the heart can be rotated and moved toward the anterior incision using tapes (arrows) encircling aorta, inferior caval vein and left pulmonary veins for exposure of coronary targets at lateral and inferior left ventricular wall. *CPB* cardiopulmonary bypass

When the heart was arrested and decompressed completely, left pulmonary veins und inferior vena cava were encircled with separate tapes*.* Traction on these tapes and rotating the heart enabled to reach all left ventricular territories by reducing the distance from skin incision to coronary arteries significantly to less than 10 cm at maximum (Fig. 1).

**Fig. 2:**
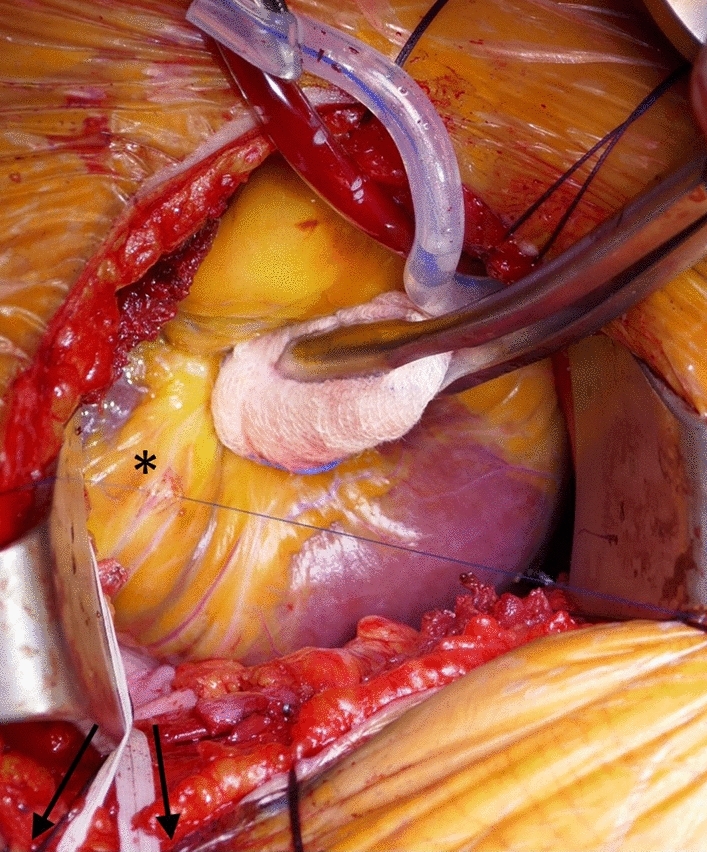
The inferior left ventricular wall is rotated toward the thoracic incision pulling tapes (arrows) encircling the caval and pulmonary veins (traction maneuver). Stable exposure is achieved for preparation, incision, and anastomosing of the PDA (*) with conventional surgical techniques. *PDA* posterior descending artery of the right coronary artery

Coronary artery target sites, especially the right coronary artery (RCA) and circumflex (CX) territories at the lateral and inferior left ventricular wall, could be exposed for finger palpation and assessment. This traction maneuver also enabled adequate and stable exposure even for extensive and complex coronary preparation if necessary as well as conventional manual knotting of all anastomotic sutures irrespective of ventricular localization (Fig. 2).

Coronary anastomoses were performed with standard anastomotic technique of running 8-0 polypropylene sutures and with usual coronary surgical instruments. First, the RA conduit was anastomosed to the most distal target (branches of RCA territory or distal CX) in an end-to-side fashion followed by sequential anastomoses to the more proximal targets in a side-to-side fashion. The LIMA was anastomosed as in situ graft to the LAD, then the RA conduit was anastomosed to the LIMA as composite T-graft or Y-graft. The last anastomosis was performed with the heart still arrested. All grafts were assessed intraoperatively using transit time flow measurement (QuickFit TTFM, Medistim, Deisenhofen, Germany).

## Results

Mean patient age was 65.9 ± 9.2 years, two patients were older than 80 years. Massive obesity (BMI > 35 kg/m^2^) was present in 3 patients. Six patients presented with non-ST elevation myocardial infarction, whereas chronic coronary syndrome was present in the remaining. Mean left ventricular ejection fraction (EF) was 48.8 ± 12.3, and EF was severely reduced (EF < 25%) in 2 patients. According to STS-Score [8], 2 patients were at intermediate (STS-score 4–8) or high (STS-score > 8) operative risk, respectively. Baseline and cardiovascular characteristics are given in Table 1.

**Table 1:** Baseline parameters

	Intervention group, *n* = 20
Baseline parameters	
Age (years)	65.9 (± 9.2)
> 80 years	2 (10%)
BMI (kg/m^2^)	27.8 (± 4.2)
Massive obesity (BMI > 35)	3 (15%)
Hypertension	16 (80%)
Diabetes mellitus	9 (45%)
Cardiovascular parameters	
Symptoms:	
CCS 1	0 (0%)
CCS 2	2 (10%)
CCS 3	15 (75%)
CCS 4	3 (15%)
2-vessel disease	6 (30%)
3-vessel disease	14 (70%)
Left main stenosis > 50%	10 (50%)
Left ventricular ejection fraction	48.8 (± 12.3)
EF < 25%	2 (10%)
NSTEMI	30%
STS-Score	1.6 (± 2)
Intermediate risk (STS-score 4–8)	1 (5%)
High risk (STS-score > 8)	1 (5%)

Data are presented as mean (± standard deviations) or absolute values (percentage %)

*BMI* body mass index, *CCS* Canadian Cardiovascular Society, *EF* ejection fraction, *NSTEMI* non-ST-elevation myocardial infarction, *STS* Society of Thoracic Surgeons

In all patients, both LIMA and RA conduits were exclusively used. A total of 65 distal anastomoses in 20 patients were performed. The average number of distal anastomoses was 3.3 ± 0.7. In 17 patients, ≥ 3 coronary arteries were anastomosed. The LAD coronary artery territory was grafted in all patients, the CX territory in 17 patients and the RCA territory in 14 patients. In 1 patient, a complex coronary reconstruction (open local coronary endarterectomy and reconstruction with patch) was performed due to diffuse sclerosis of the coronary target. In another patient, an occluded right coronary artery was investigated during the operation but found to be too small and diffusely diseased to be grafted. All other coronary targets preoperatively recommended by the heart team to be re-vascularized could be anastomosed, thus complete anatomical revascularization was achieved in all but one patient. Operative data are given in Table 2.

**Table 2:** Operative data

Conduits used	
LIMA	20 (100%)
RA	20 (100%)
Revascularization territory of	
LAD	20 (100%)
RCX	17 (85%)
RCA	13 (65%)
Combinations	
LAD + RCX + RCA	10 (50%)
LAD + RCx	7 (35%)
LAD + RCA	3 (15%)
Number of distal anastomoses	3.3 (± 0.7)
2	3 (15%)
3	9 (45%)
4	8 (40%)
Coronary thrombendarterectomy	1 (5%)
Length of surgery (minutes)	316 ± 37
CPB time (minutes)	159 ± 29
Aortic cross-clamp time (minutes)	98 ± 22

Data are presented as mean (± standard deviations) or absolute values (percentage %)

*LIMA* left internal mammary artery, *RA* radial artery, *LAD* left anterior descending artery, *RCX* ramus circumflexus, *RCA* right coronary artery, *CPB* cardiopulmonary bypass

**Fig. 3:**
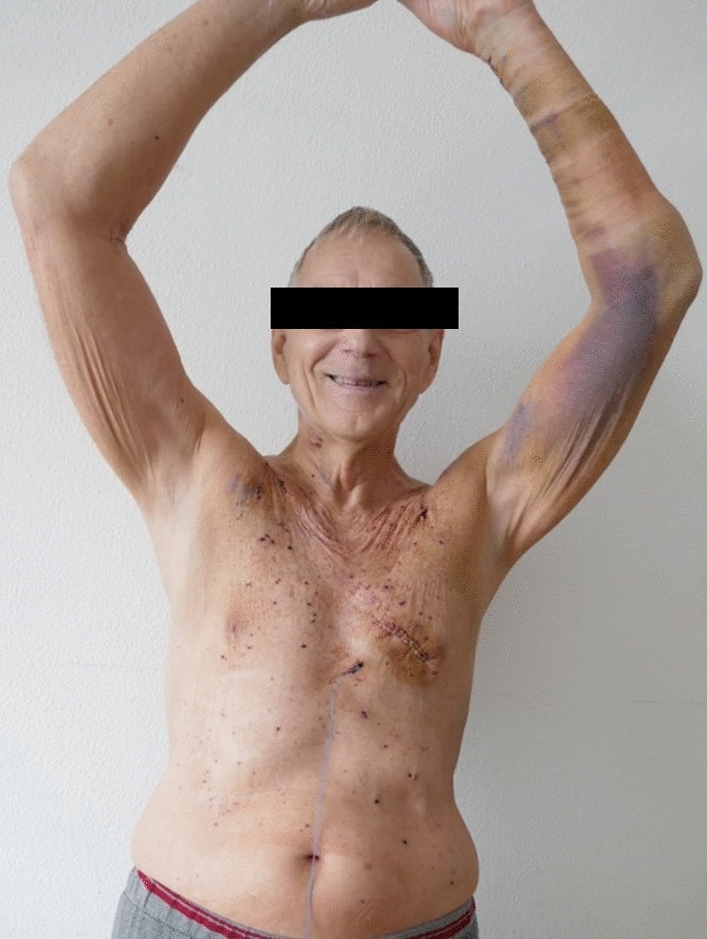
74-year-old patient at postoperative day 5 (LIMA to LAD, RA to CX and PDA) elevating both arms above the head to demonstrate thoracic stability. *LIMA* left internal mammary artery, *LAD* left anterior descendens, *RA* radial artery, *CX* circumflex artery, *PDA* posterior descending artery

There was no hospital mortality, no myocardial infarction, no repeat revascularization, no stroke. Complications were low. ICU stay was 1 day in 17 patients. Avoiding sternotomy, preserved thoracic stability allowed immediate mobilization of all patients (Fig. 3). 14 patients left the hospital within 8 days after the operation. In-hospital outcome is given in Table 3.

**Table 3:** Postoperative adverse events and outcome

Adverse events	
Low cardiac output	1 (5%)
Myocardial infarction	0 (0%)
Revision due to bleeding	2 (10%)
Dialysis	0 (0%)
Delir	2 (10%)
Pneumonia	2 (10%)
New onset of atrial fibrillation	7 (35%)
Superficial wound infection	1 (5%)
Stroke	0 (0%)
Outcome parameters	
Time on ICU (days)	1.3 (± 0.7)
≤ 1 day	17 (85%)
In-hospital stay (days)	8.7 (± 3.1)
≤ 8 days	14 (70%)
In-hospital mortality	0 (0%)

Data are presented as mean (± standard deviations) or absolute values (percentage %)

*ICU* intensive care unit

## Discussion

In this pilot series of 20 unselected patients, minimally invasive CABG via left anterior thoracotomy using only arterial grafts was technically feasible. In-hospital outcomes were favorable with swift mobilization and early recovery.

The small thoracic incision is located more anteriorly compared to a more lateral approach used in other MICS CABG [3, 4, 9–11]. This technical detail is important because distance from the incision to both aorta as well as coronary target sites becomes shorter. Peripheral insertion of CPB cannulas and transthoracic aortic cross-clamping enables an unrestricted direct vision of the operation field through a small thoracic incision. Transthoracic cross-clamping from the left side was possible by pulling the ascending aorta with an encircling tape to the left. With the heart arrested and emptied, the working space inside the pericardium becomes much larger while hemodynamics remains stable. In combination with the traction maneuver, a stable exposure of lateral and inferior wall coronary targets at a distance less than 10 cm can be established. Direct examination of coronary artery target sites even by palpation and use of common surgical instruments are possible.

The main advantage of the proposed technique [5] obviously results from the avoidance of sternotomy. Enhanced thoracic stability, reduced wound size and reduced wound infection risk associated to a minimally invasive thoracotomy approach have already been shown to result in accelerated early recovery and return to normal physical activity in different cardiac surgery procedures [12].

Minimally invasive CABG is often a compromise between surgical trauma and complete revascularization, and the number of distal anastomoses performed in surgical revascularization of patients with multi-vessel disease is considered as an indirect parameter for the completeness of revascularization [13]. The number of distal anastomoses in this initial pilot series compares well to the average number of 3.2 reported for conventional CABG in the recent German Heart Surgery Report 2018 [14], but it clearly exceeds that reported in MICS CABG [3, 4, 9–11].

A composite graft technique with LIMA and RA in a Y- or T-graft constellation [15] was exclusively applied in all patients enabling a complete anatomical revascularization in the vast majority of patients in this initial experience. With such graft constellation, special attention has been directed to sufficient length of the radial artery, especially in patients with dilated hearts associated to cardiomyopathy. In case of inappropriate radial artery length, graft strategy must be adapted intraoperatively, e.g., an additional venous graft can be anastomosed to the most distal coronary target [5]. However, this was not necessary in this small pilot series.

Benefit from surgical revascularization is most pronounced in multi-vessel disease patients with diffuse coronary disease (high Syntax score), diabetes mellitus, and ischemic cardiomyopathy (low left ventricular ejection fraction) [7]. While especially such patients often were excluded from minimally invasive techniques [4], we included patients with diffuse coronary disease, low ejection fraction, high surgical risk, massive obesity or compromised lung function in this pilot series. Which patients may benefit most from the presented technique has to be investigated in further studies.

It may be of concern that duration of the operation was clearly longer than that known from routine CABG. However, this did not translate into an increased length of ICU or hospital stay. Additionally, gaining more experience it might be possible to shorten the procedure distinctly as it has been shown for newly introduced surgical techniques previously [11].

Aortic manipulation and CPB are associated with an increased stroke rate. To minimize this risk, we routinely performed a preoperative CT scan [16]. In case of atherosclerosis distal to the ascending aorta, a central cannulation via the right subclavian artery was preferred to avoid retrograde perfusion. In case of atherosclerotic disease of the ascending aorta, alternative no-touch aortic CABG techniques should be preferred. Whether such algorithm is effective in minimizing stroke risk remains to be investigated in future. In this pilot series, though limited by its small size, no stroke was observed.

In conclusion, totally arterial multi-vessel coronary revascularization can be performed minimally invasive avoiding sternotomy in a non-selected all-comer cohort with complete anatomical revascularization. Respecting sternal integrity might considerably improve both patients and physician’s acceptance of surgical myocardial revascularization.
